# Implant surface selection in primary cosmetic breast augmentation: A national cross-sectional study of UK plastic surgeons

**DOI:** 10.1016/j.jpra.2025.06.004

**Published:** 2025-06-17

**Authors:** Chloe Jordan, Krzysztof Sosnowski, Rushabh Shah, Charles Malata

**Affiliations:** a Department of Plastic and Reconstructive Surgery, Addenbrooke’s Hospital, Cambridge University Hospitals NHS Foundation Trust, Hills Rd, Cambridge, United Kingdom; b School of Clinical Medicine, University of Cambridge, Hills Rd, Cambridge, United Kingdom; c Cambridge Breast Unit, Addenbrooke’s Hospital, Cambridge University Hospitals NHS Foundation Trust, Hills Rd, Cambridge, United Kingdom; d Anglia Ruskin University School of Medicine, Anglia Ruskin University, East Rd, Cambridge, United Kingdom

**Keywords:** Breast augmentation, BIA-ALCL, Cosmetic surgery, Implant selection, Survey, Breast implants

## Abstract

**Introduction:**

Breast augmentation remains one of the most common cosmetic surgical procedures globally yet implant surface preference (textured versus smooth) remains a key area of debate. Whilst textured implants have traditionally been favoured in the UK and Europe, concerns around breast implant-associated anaplastic large cell lymphoma (BIA-ALCL) and the absence of formal guidelines have contributed to wide variations in practice. This study therefore examines current UK practice among plastic surgeons, focusing on surgeon preferences and the factors guiding decision-making.

**Methods:**

An anonymised online survey was distributed to UK consultant plastic surgeons performing cosmetic breast augmentation. Data collected included implant surface, brand, incision, pocket, case volume, and ranked decision-making factors. Free-text responses were analysed thematically. Descriptive statistics and chi-square testing were used to explore associations.

**Results:**

Seventy-five consultant plastic surgeons responded (response rate = 31.3 %). Textured implants were most commonly used (82.7 %), although smooth implants were more frequently selected by high-volume surgeons (>50 cases/year, χ²(5) = 11.79, *p* = 0.038, Cramér’s *V* = 0.37). Mentor was the preferred brand (53.3 %), while Motiva was more commonly selected by smooth implant users (χ²(5) = 58.3, *p* = 0.011, Cramér’s *V* = 0.29). All surgeons preferentially used inframammary incisions; subglandular placement was most common (54.7 %). The top four decision-making factors in implant surface selection were patient preference, cosmetic outcome, risk of capsular contracture and BIA-ALCL possibility. Qualitative themes highlighted the influence of patient expectations, safety concerns, and institutional policy.

**Conclusion:**

UK practice in cosmetic breast augmentation remains varied. Although most surgeons preferred textured implants, a shift towards smooth implants appears to be emerging among high-volume operators.

## Introduction

Breast augmentation remains one of the most commonly performed cosmetic surgical procedures worldwide, with an estimated 1.5 million women undergoing breast implant surgery annually. In the United Kingdom (UK), approximately 7,000 cases were reported in 2022, underscoring its ongoing relevance within UK plastic surgery practice. ¹ As patient expectations and surgical techniques have evolved, so too have implant technologies. Modern breast implants vary in gel cohesiveness, anatomical shape, and surface characteristics, particularly in the degree of shell texturing.² These modifications aim to reduce complication rates and improve aesthetic outcomes, however consensus on optimal implant design remains elusive.

There is notable geographical variation in implant preferences. In the United States, smooth, round gel implants remain the preferred choice, while in the UK, approximately 85 % of surgeons favour textured, anatomically shaped gel implants. ³ This discrepancy likely reflects differences in training, clinical tradition, perceived safety profiles, and potential litigatory implications. The smooth-versus-textured debate continues to divide opinion, as each surface type carries a distinct risk-benefit profile. At present, no universally accepted guidelines exist to support surgeons in selecting the most appropriate implant on an individual patient basis.

Despite advancements in implant design, complications remain an ongoing challenge, some of which require revision surgery. Capsular contracture is the most frequently reported long-term complication, affecting up to 15 % of cosmetic implants.⁴ Textured implants were originally developed to address this issue, under the hypothesis that surface roughness would reduce capsule contraction.^5^, ^6^, ^7^ However, empirical evidence has been mixed, with some studies demonstrating no clear benefit over smooth implants. Furthermore, textured implants have been linked to higher rates of infection, bacterial biofilm formation, and more recently, breast implant-associated anaplastic large cell lymphoma (BIA-ALCL), a rare but potentially life-threatening T-cell lymphoma.^8^^,^^9^

As concerns around BIA-ALCL have grown, regulatory scrutiny has intensified. Several textured implant models have been voluntarily withdrawn or restricted in multiple countries. Nonetheless, clinical practice remains highly variable. Surgeons differ in their choice of implant brand, surface texture, and operative technique, with decisions influenced by individual training, local norms, manufacturer availability, and institutional policies. In some cases, hospitals have restricted the use of specific implants, further contributing to variation in care.

This ongoing heterogeneity highlights the need for updated, UK-specific data to inform evidence-based practice. While several surveys have explored implant use trends abroad, there is a paucity of data examining current practice in the UK. In the absence of formal guidelines, surgeons are left to navigate this evolving field based largely on experience and preference, rather than consensus. The aim of this study is to examine national practice patterns among UK consultant plastic surgeons performing primary cosmetic breast augmentation, with a focus on the use of smooth versus textured implants. We explore implant selection trends, geographical and institutional variation, surgeon case volumes, and the factors influencing decision making. By addressing this gap, this study aims to inform evidence-based clinical guidelines and contribute to safer, more standardised patient care in aesthetic breast surgery.

## Methods

### Questionnaire design and distribution

A national online cross-sectional survey was developed using Google forms and distributed electronically to all substantive UK based plastic surgery consultants listed within the membership database of the British Association of Plastic, Reconstructive and Aesthetic Surgeons (BAPRAS). Only those surgeons who perform primary breast augmentation within their practice and are full members of BAPRAS were included. Full members who were retired, or based outside the UK were excluded from our study.

The survey questions (Appendix A) covered the following six domains:

1. General demographics: Name of unit, geographical region, and subspecialty interest within NHS practice.
2. Workload volume: Number of cosmetic breast augmentations performed privately per year.
3. Implant preferences: Type of implant used most frequently (smooth vs. textured), preferred brand, and implant pocket.
4. Surgical techniques: Most commonly used incision type.
5. Factors influencing implant choice: Ranking of factors (e.g., capsular contracture risk, rotation risk, ALCL risk) that determine the choice of smooth vs. textured implants.
6. Additional comments: Open-ended responses regarding factors affecting implant selection not covered in the survey.

The survey was distributed electronically, via email and the BAPRAS monthly bulletin (March edition), with responses collated between 24th February 2025 and 21st April 2025. The survey was designed to be concise, consisting of 9 specific questions, and required approximately 3 min to complete. Participation was voluntary, and all responses were anonymised. Surgeons who did not respond to the initial email were sent follow up email reminders on three separate occasions, to maximise response rate, as per the Dillman method.^10^

### Data collection and analysis

Data from all responses were anonymised and included in the final analysis. Microsoft Excel (Office 2019, Microsoft Corp., Redmond, WA) was used for initial review and cleaning.

Statistical analyses were performed using SPSS (SPSS Inc., Chicago, Illinois, USA). Descriptive statistics summarised the demographic and clinical practice data, while chi-square tests were employed to identify significant associations between categorical variables such as implant preference and annual surgical volume, region, and implant pocket selection. Statistical significance was defined as *p* < 0.05. Regional subgroup analyses were only performed for regions with five or more respondents, as comparisons below this threshold were considered statistically unreliable.

### Statistical considerations

This was an exploratory, hypothesis-generating study designed to describe current UK practice. Formal a priori power calculations were not performed. However, subgroup comparisons were interpreted with caution, and chi-square analyses were restricted to comparisons where expected cell counts were >5 to preserve statistical validity.

We acknowledge that multiple comparisons across variables (e.g., region, surgical volume, implant brand) may increase the risk of Type I error. Given the descriptive nature of the study, no formal corrections (e.g. Bonferroni or Benjamini-Hochberg) were applied, though results are interpreted conservatively. For statistically significant chi-square associations, Cramér’s V was calculated and reported to provide additional context regarding effect size.

### Ethical considerations

No formal ethical approval was required for this survey, as it involved anonymised data collection from healthcare professionals. Participation was voluntary, and implied consent was obtained through completion of the survey.

## Results

### Demographics

A total of 75 consultant plastic surgeons completed the survey (response rate 31.2 %). Respondents were drawn from all major UK regions, with London and the Southeast being the most represented (*n* = 11 each). Participants reported a range of NHS subspecialty interests, with the majority specialising in breast reconstruction (*n* = 36). The most commonly reported annual volume of cosmetic breast augmentations was 10–20 cases (Table 1).

**Table 1:** Surgeon demographics (*n* = 75).

Geographical region	*N* (%)
East Midlands^a^	2 (2.7 %)
East of England	6 (8 %)
London	11 (14.7 %)
North East	7 (9.3 %)
North West	7 (9.3 %)
Northern Ireland^a^	1 (1.3 %)
Scotland	8 (10.7 %)
South East	11 (14.7 %)
South West	7 (9.3 %)
Wales^a^	1 (1.3 %)
Wessex^a^	1 (1.3 %)
West Midlands	6 (8 %)
Yorkshire and the humber	7 (9.3 %)

NHS subspecialty interest	N (%)

Breast reconstruction	36 (48)
Hands	13 (17.3)
Skin	8 (10.7)
Cleft	2 (2.7)
Trauma/lower limb	2 (2.7)
Other (Sarcoma, Burns, Microsurgery)	5 (6.7)
Solely private practice	8 (10.7)

a Regions with <5 respondents were excluded from subgroup statistical analysis.

#### Implant surface preference

Implants were categorised as either smooth or textured, with all microtextured and macrotextured responses grouped into the textured category. Textured implants were most commonly preferred, reported by 82.7 % (*n* = 62) of surgeons, while 17.3 % (*n* = 13) favoured smooth implants.

**Figure 1:**
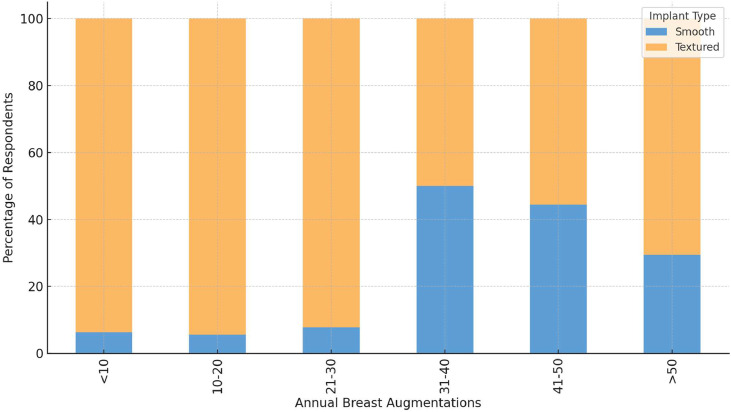
Implant surface preference by annual surgical volume of breast augmentations (smooth vs textured implants).

There was a statistically significant association between implant surface preference and annual surgical volume (χ²(5) = 11.79, *p* = 0.038, Cramér’s *V* = 0.37), suggesting that high-volume surgeons (>50 cases/year) were more likely to favour smooth implants (Figure 1).

#### Implant brand

Mentor was the most frequently preferred implant brand (53.3 %, *n* = 40), followed by Motiva (24 %, *n* = 18). Other brands included Nagor (9.3 %, *n* = 7), GCA (6.7 %, *n* = 5), Polytech (2.7 %, *n* = 2), and Sebbin (2.7 %, *n* = 2).

**Figure 2:**
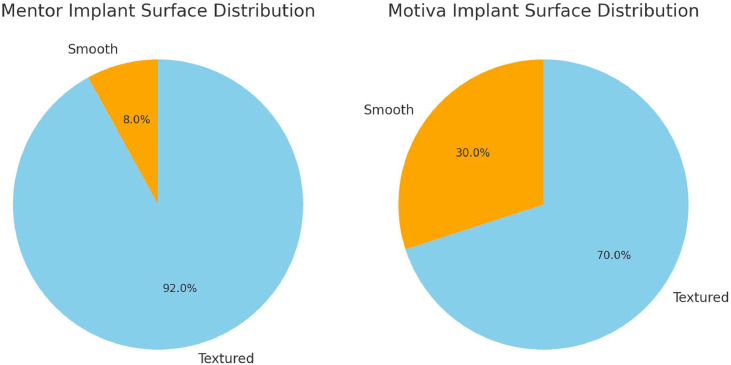
Distribution of implant surface type (smooth vs textured) amongst surgeons who preferred Mentor (left) and Motiva (right) implants.

There was a statistically significant association between implant brand preference and implant surface selection (χ²(5) = 58.3, *p* = 0.011, Cramér’s *V* = 0.29). Surgeons using smooth implants more commonly favoured Motiva, whereas Mentor was the predominant choice amongst surgeons preferring textured implants (Figure 2).

#### Annual surgical volume

The distribution of annual case volumes was varied. The most common category was 10–20 augmentations per year (24 %, *n* = 18), followed by:

• >50 cases: 23 % (*n* = 17)
• <10 cases: 21.3 % (*n* = 16)
• 21–30 cases: 17.3 % (*n* = 13)
• 41–50 cases: 12 % (*n* = 9)
• 31–40 cases: 2.7 % (*n* = 2)

#### Surgical technique

All respondents reported using an inframammary incision. The most common implant pocket was subglandular (54.7 %, *n* = 41), followed by dual-plane (36 %, *n* = 27), and subpectoral placement (9.3 %, *n* = 7).

Among surgeons preferring the subglandular pocket, 85.4 % (*n* = 35) used textured implants and 12.2 % (*n* = 5) used smooth. Among dual-plane users, 81.5 % (*n* = 22) used textured and 11.1 % (*n* = 3) used smooth. No statistically significant associations were observed between implant surface preference and pocket choice (χ²(2) = 1.40, *p* = 0.50).

#### Regional variation

**Figure 3:**
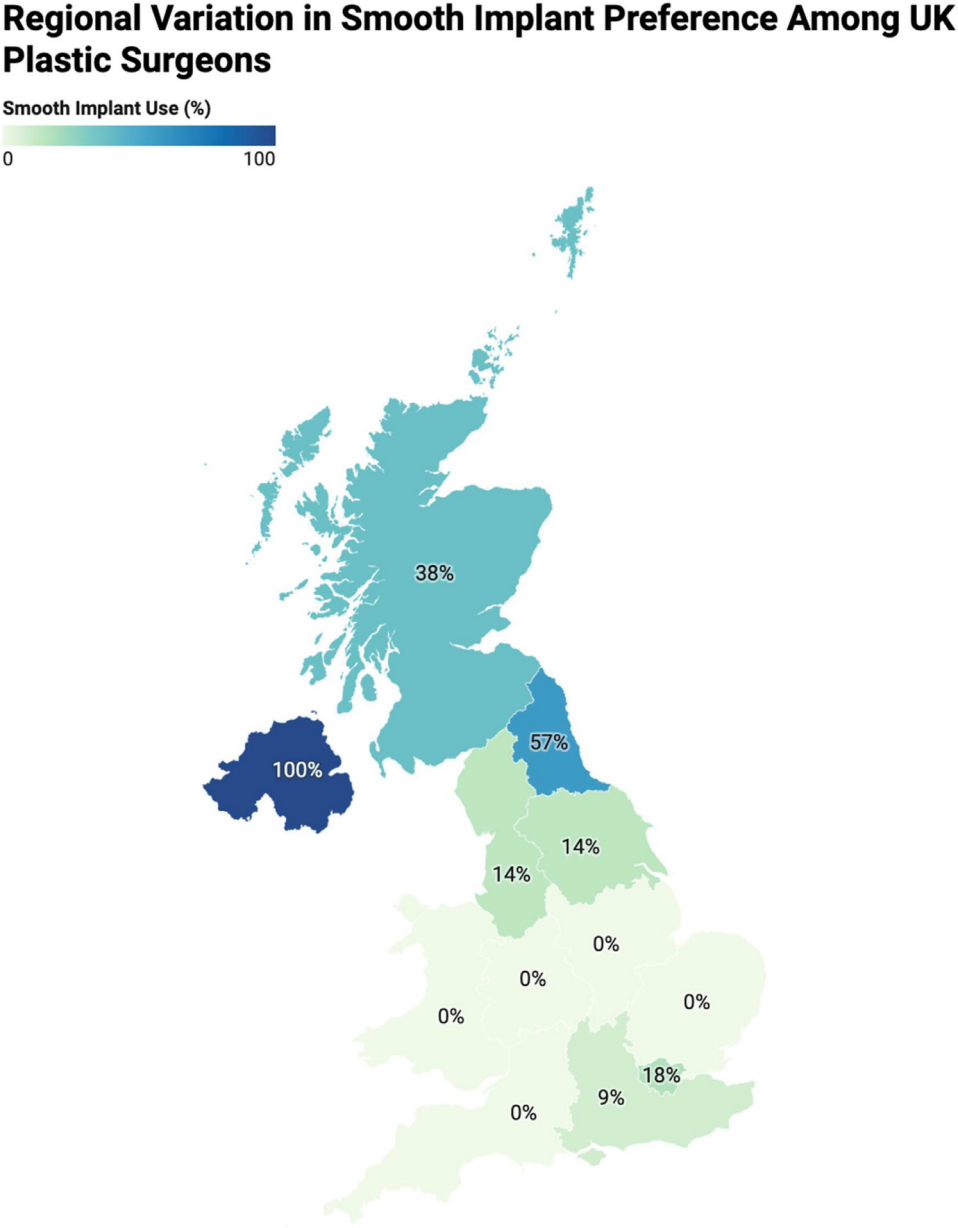
Chloropleth map demonstrating geographic distribution of smooth implant preference in the UK.

Textured implants remained the predominant choice across all regions. Smooth implant usage was most frequently reported in the North-East (*n* = 4), followed by Scotland (*n* = 3), London (*n* = 2), Yorkshire, Northern Ireland, South-East and South-West (*n* = 1 each) (Figure 3). No statistically significant regional differences in implant choice preference were observed (χ²(8) = 12.02, *p* = 0.250).

#### Factors influencing implant choice

Surgeons ranked six factors influencing implant choice on a scale of 1 (most important) to 6 (least important). The three highest-ranked factors were:

• Patient preference–Mean: 2.04 ± 1.28
• Cosmetic outcome–Mean: 2.45 ± 1.22
• Risk of capsular contracture–Mean: 2.73 ± 1.29

Factors ranked as less important included:

• Risk of BIA-ALCL–3.67 ± 1.15
• Advice from medical indemnity providers–4.56 ± 1.08
• Cost–4.61 ± 1.03

**Figure 4:**
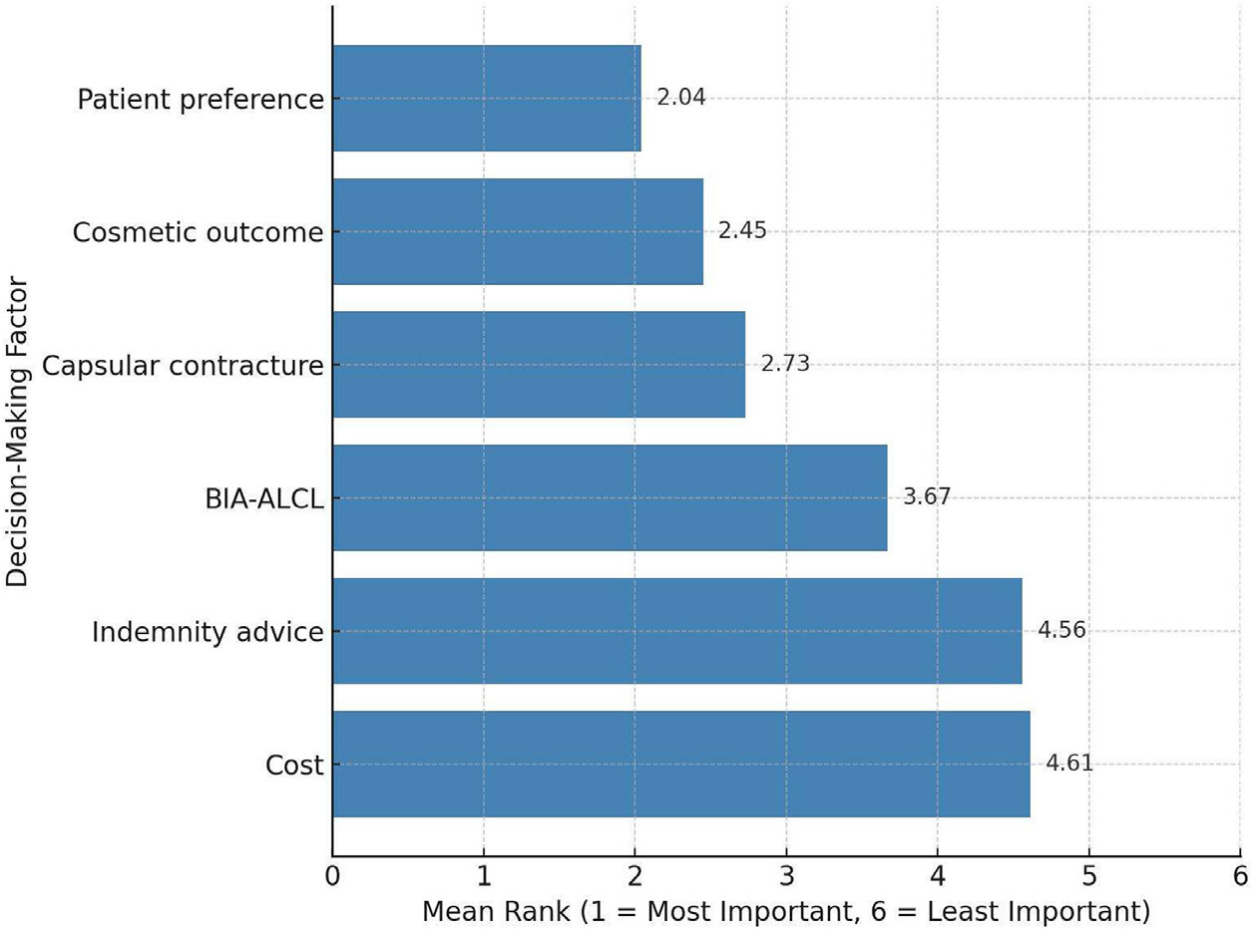
Mean ranking of factors influencing implant surface selection among UK plastic surgeons.

These findings are summarised visually in Figure 4.

#### Qualitative analysis of free-text comments

Several respondents offered free-text comments, providing additional insight into implant selection. Four key themes were identified:

• Patient involvement: Many surgeons described involving patients in the final decision, particularly where there was no strong clinical preference. Some noted that patients were increasingly aware of BIA-ALCL and would specifically request smooth implants, while others preferred textured based on prior experience or research.
• Safety concerns: A number of respondents reported moving away from textured implants due to safety concerns. Others continued to use textured implants but were more selective, balancing perceived risks with specific case factors.
• Institutional or indemnity influence: Several surgeons commented that implant choice was dictated or influenced by hospital policy or advice from indemnity providers, in some cases limiting access to certain devices or prompting a shift to smooth implants.
• Aesthetic and technical considerations: Some surgeons continued to prefer textured implants in specific scenarios, citing better control over implant positioning, upper pole fullness, and reduced risk of malposition, particularly in more complex or borderline indications.

## Discussion

This national cross-sectional study provides an up-to-date overview of primary cosmetic breast augmentation practice amongst UK plastic surgeons, with a focus on implant surface selection. The results highlight significant variation in practice, both in terms of implant choice and surgical approach. Despite growing awareness of BIA-ALCL and increasing regulatory scrutiny surrounding textured devices, they remain the favoured choice among British plastic surgeons. Evidence suggests the current risk of BIA-ALCL in the UK is around 1 in 24,000 implants, with no confirmed cases reported in patients with smooth implants.^11^^,^^12^ Regardless, most UK plastic surgeons favour textured implants for their perceived advantages.^13^ Our results reflect this conundrum: although the majority of UK plastic surgeons prefer textured implants, there is an emerging shift towards the use of smooth implants among high-volume surgeons, perhaps reflecting greater exposure to emerging safety data, shifting patient expectations and evolving industry trends.

Significant geographic variation in implant preferences has been well documented. In the United States, most surgeons have long favoured smooth, round implants, whereas in Latin America, Asia and Europe a large majority use textured devices.^14^ Additionally, a recent survey by Jabir et al.,^13^ found textured implants to be widely used in the UK and Ireland, but reported an increase in smooth implant use compared to previous years, aligning with our results. The accumulating evidence and guidance related to BIA-ALCL risk, as well as international influence, likely accounts for this gradual shift towards smooth implants.^15^^,^^16^

The ongoing use and popularity of textured implants within the UK likely reflects a combination of numerous factors: historical familiarity, perceived benefits of aesthetic superiority, and confidence in long-term outcomes, attributed to a favourable complication profile. These implants have traditionally been valued for their reduced rates of rotation and malposition.^17^ Additionally, many surgeons associate texturing with improved upper-pole control and reduced capsular contracture risk.^18^ These factors ranked among the most important decision-making factors in this survey, alongside patient preference and aesthetic outcome.

Implant brand preferences appeared to mirror the trends noticed in implant surface preference. Mentor, a company historically associated with textured implants, was the most frequently used implant brand (over half of surgeons), followed by Motiva (a newer brand specialising in nano-textured devices). This finding reinforced the role of training exposure and brand familiarity in surgeon preference. Many surgeons remain loyal to the brands and types of implants they have trained with or which their institution stocks, however with the introduction of newer implant lines, some surgeons have started adopting these alternatives, often times in parallel with their continued use of well-established textured implants.

With regards to surgical technique, our results highlight a consensus. All respondents preferred the inframammary incision, consistent with global and national trends.^11^^,^^13^ This approach has the advantages of direct pocket visualisation, good surgical access, and a well-hidden, aesthetically favourable scar. The choice of implant pocket, however, was more varied, with over half our respondents preferring subglandular placement, followed by dual-plane and subpectoral techniques. Although subglandular placement may reduce overall operative time and postoperative pain, it is associated with higher capsular contracture rates, especially with smooth implant use, when compared to subpectoral or dual-plane placement.^19^ Regardless, the choice of implant pocket is complex, and surgeons must balance the risk of capsular contracture with other considerations such as soft tissue coverage, aesthetic goals and likelihood of animation deformity. Consequently, some surgeons have advocated the use of subfascial placement as a middle ground, as it offers lower contracture rates and reduced visibility without muscle distortion.^20^^,^ ^21^ There have also been a recent resurgence in popularity of Tebbetts’ dual-plane technique, as it combines the benefits of subpectoral and subglandular positioning, though it does have drawbacks such as potential for animation deformity.^22^ Although our study did not directly assess the reasons behind pocket selection, qualitative comments reinforced the importance of tailoring these decisions to the patient, considering their anatomy, tissue characteristics and expectations.

When considering factors which influence implant selection, our findings highlight the central role of patient involvement. Patient preference was ranked as the most important decision-making factor, followed by desired cosmetic outcome, and minimising risk of capsular contracture. Although the risk of BIA-ALCL was acknowledged, it was not within the top three considerations, demonstrating that oncologic risk is one part of a broader decision-making framework for the majority of UK plastic surgeons. Interestingly, non-clinical factors such as cost or advice from medical indemnity providers were less commonly ranked as important, though a few respondents did note that procurement restrictions or institutional guidance influenced their choice of implant selection.

Medicolegal considerations also appear to shape implant choice in certain settings. While our survey did not identify a significant association between workplace setting and implant preference, qualitative responses highlighted that institutional procurement policies and indemnity advice may influence decision-making. Some surgeons noted restricted access to certain textured implants within NHS Trusts, or guidance from indemnity providers prompting more cautious implant selection. These non-clinical factors, though not formally quantified in this study, reflect the evolving medicolegal landscape surrounding breast implants. As awareness of BIA-ALCL increases and regulatory scrutiny continues, the role of indemnity bodies and institutional policy may exert growing influence on implant availability and surgeon choice.

The evolving regulatory landscape in the UK has significantly shaped current implant use trends. The PIP implant scandal in 2010, which exposed widespread use of substandard silicone implants, had a lasting impact on surgeon trust, patient perception, and institutional policy.^23^ This event prompted stricter oversight from the MHRA and highlighted the need for robust national surveillance systems. In response, the Breast and Cosmetic Implant Registry (BCIR) was launched in 2016 to track implant usage and facilitate traceability in the event of safety concerns.^24^ Furthermore, MHRA guidance regarding the association between textured implants and breast implant-associated anaplastic large cell lymphoma (BIA-ALCL) has reinforced caution in implant selection. This backdrop of regulatory scrutiny and public concern continues to influence surgeon decision-making, even in the absence of new safety alerts during our survey period.

Analysis of the qualitative responses offered an insight into the increasing influence of media, litigation and online patient forums in shaping patient views. Numerous surgeons highlighted that patients were arriving to pre-operative consultations with pre-formed opinions, often influenced by widely publicised BIA-ALCL stories. With increasingly easy access to digital information, patient counselling is becoming more nuanced. Many surgeons described how they now use implant consultations as an opportunity to reframe discussions around safety and satisfaction–acknowledging patient safety concerns, while guiding choice with evidence and professional clinical judgement.

### Strengths and limitations

This study benefits from a high number of responses from a broad range of consultants across UK regions and subspecialities, with most performing over 10 augmentations annually. This provides a representative overview of current practice among UK plastic surgeons undertaking primary cosmetic breast augmentation. While the 31.2 % response rate may appear modest, it aligns with accepted benchmarks for web-based surveys and was improved through repeated follow-up reminders.^25^ Additionally, the study design incorporated both quantitative and qualitative data, helping to provide both measurable trends and contextual nuance, offering a more complete picture about the factors implicated in the decision-making process.

However, several limitations must be considered. Firstly, the survey relied on self-reported data, potentially introducing recall bias, an issue innate to all survey-based studies. While the overall response rate was in line with accepted benchmarks, we acknowledge the possibility of non-response bias. Due to the anonymous nature of the survey, we were unable to compare the geographic or demographic profiles of responders versus non-responders. It is therefore possible that those who chose to respond may differ systematically in their implant preferences or practice patterns, potentially limiting the generalisability of our findings. Secondly, our survey included only consultant plastic surgeons affiliated with BAPRAS and did not include the wider group of practitioners performing cosmetic breast augmentation in the UK. Surgeons from aesthetic or general surgery backgrounds, such as members of BAAPS, ABS, or GMC-registered private cosmetic practitioners, may differ in their implant preferences. Future surveys should aim to include these groups to provide a more comprehensive overview of national practice. Finally, while this survey offers valuable cross-sectional insight, it does not capture long term trends or outcomes. National registries such as the Breast and Cosmetic Implant Registry (BCIR) may play an important future role in capturing real-world data and supporting guideline development.^26^

As of now, no new MHRA safety alerts, recalls, or major implant-related publications appear to have been issued during the survey period (24th February–21st April 2025), based on available records from the MHRA and implant registry announcements. However, the background climate of regulatory caution and ongoing media coverage surrounding BIA-ALCL remains relevant and may have influenced surgeon sentiment. Indeed, this climate formed part of the original motivation for conducting this study, as we anticipated that heightened awareness of safety concerns might be prompting changes in implant selection practices across the UK.

## Conclusion

This study is the first national survey focusing specifically on implant surface preferences in primary cosmetic breast augmentation among UK plastic surgeons. It provides valuable insight into current practice patterns and updates the limited data previously available on UK trends. Although textured implants remain the most widely used, there is clear evidence of a shift toward smooth implants, particularly among high-volume surgeons. Decision-making is influenced by a combination of patient preference, aesthetic outcomes, and perceived risk of complications such as capsular contracture. Although awareness of BIA-ALCL is growing, it remains one of several factors considered during implant surface selection, rather than a sole driver. By clarifying current trends and the rationale behind them, this study can help inform evidence-based guidelines and encourage further research to optimise patient outcomes in cosmetic breast augmentation.

## Funding

None.

## Ethical approval

Not required.

## Conflicts of interest

None declared.

## Appendix Supplementary materials

Image, application 1
