# Supplementary material for: Implant surface selection in primary cosmetic breast augmentation: A national cross-sectional study of UK plastic surgeons
Source: JPRAS Open. 2025 Jun 17;45:212–24. doi: 10.1016/j.jpra.2025.06.004 (PMC12270748; doi:10.1016/j.jpra.2025.06.004)
Supplement: Supplementary file 1 [file mmc1.pdf]

National survey on the use smooth vs textured implants in primary cosmetic breast augmentation.

Dear Colleague

We would like to invite you to participate in our national survey on the use of smooth vs textured implants in primary cosmetic breast augmentation.

We believe there is significant heterogeneity in practice amongst colleagues undertaking cosmetic breast augmentation, with a perceived shift towards the use of smooth implants.

Our aim is to explore national practices through the data collected in this survey. We seek to describe the workload volume, outline variation across regions and explore the factors that are key to determining the choice of smooth vs textured implant.

Our survey is entirely anonymous, consists of 9 specific questions and requires only 3 minutes of your time to complete. We hope you are able to participate, and thank you in advance for taking the time to answer the questions.

With best regards,

Professor Charles Malata (Consultant Plastic and Reconstructive Surgeon), Mr Rushabh Shah (Specialist Trainee in Plastic Surgery), Miss Chloe Jordan (Core Surgical Trainee), Mr Krzysztof Sosnowski (Medical Student)

* Indicates required question

1. Email *

____

2. Name of Unit *

____

3. Geographical region *

Mark only one oval.

- South West
- West Midlands
- London
- North West
- Yorkshire and The Humber
- Scotland
- East Midlands
- East of England
- Wales
- Northern Ireland
- North East
- Wessex
- Isle of Man
- South East

4. Sub-speciality interest within NHS *

Mark only one oval.

- Head and Neck
- Breast reconstruction
- Sarcoma
- Hands
- Skin
- Trauma/Lower limb
- Cleft
- Paediatric
- Other: ______________________

5. How many cosmetic breast augmentations do you perform privately every year? *

Mark only one oval.

- <10
- 10-20
- 21-30
- 31-40
- 41-50
- >50

6. Which implant type do you use the most? *

Mark only one oval.

- Smooth
- Microtextured
- Macrotextured
- Other: ____________________

7. Which incision do you use the most? *

Mark only one oval.

- Periareolar
- Inframmamary
- Minimal access trans-axillary
- Minimal access trans-umbilical

8. What brand do you prefer *

Mark only one oval.

- Mentor
- EuroSilicone
- Motiva
- Sebbin
- Other: __________

9. What is your preferred implant pocket *

Mark only one oval.

- Subglandular
- Subpectoral
- Dual plane

10. Please rank the importance of the below factors in choice of implant between smooth vs textured (1- Most important, 5- Least Important) *

Mark only one oval per row.

	1	2	3	4	5	6
**Patient preference**	⬭	⬭	⬭	⬭	⬭	⬭
**Risk of capsular contracture**	⬭	⬭	⬭	⬭	⬭	⬭
**Risk of BIA-ALCL**	⬭	⬭	⬭	⬭	⬭	⬭
**Cosmetic outcome**	⬭	⬭	⬭	⬭	⬭	⬭
**Advice from medical indemnity companies**	⬭	⬭	⬭	⬭	⬭	⬭
**Cost of implant**	⬭	⬭	⬭	⬭	⬭	⬭

11. Do you have any comments about the factors affecting your decision making process with regard to implant selection, which is not covered in the question above? *

This content is neither created nor endorsed by Google.

Google Forms
